# Revealing the role of Peg13: A promising therapeutic target for mitigating inflammation in sepsis

**DOI:** 10.1590/1678-4685-GMB-2023-0205

**Published:** 2024-05-31

**Authors:** dan Wang, Zhiqiang Lin, Meixia Su, Yiqing Zhou, Mengjie Ma, Minghui Li

**Affiliations:** 1 Shaoxing People’s Hospital, Department of Infectious Diseases, Shaoxing, Zhejiang, People’s Republic of China.; 2 Shaoxing People’s Hospital, Department of general surgery, Shaoxing, Zhejiang, People’s Republic of China.

**Keywords:** Paternally expressed 13, sepsis, high mobility group box 1, interleukin-6, inflammatory response

## Abstract

To investigate the role of Peg13 in modulating the inflammatory response in sepsis, we established Lipopolysaccharide (LPS)-induced 293T cells and mouse models. Peg13 expression was assessed at various time points after infection using RT-qPCR. The levels of high mobility group box 1 (HMGB1) and interleukin-6 (IL-6) were quantified through ELISA. A total of 44 septic patients and 36 healthy participants were recruited to measure Peg13 and HMGB1 levels in the blood. Peg13 demonstrated significant down-regulation in the supernatant of LPS-induced 293T cells and in the blood of LPS-induced mice. Moreover, the levels of proinflammatory cytokines HMGB1 and IL-6 were elevated in both the supernatant of LPS-induced cell models and blood specimens from LPS-induced murine models, and this elevation could be notably reduced by Peg13 suppression. In a clinical context, Peg13 and HMGB1 levels were higher in septic patients compared to healthy subjects. Peg13 exhibited a negative correlation with HMGB1, C-reactive protein (CRP), and erythrocyte sedimentation rate (ESR) among septic patients. Peg13 mitigates the inflammatory response by reducing the release of proinflammatory cytokines HMGB1 and IL-6 in sepsis, presenting a potential therapeutic target for alleviating inflammation in sepsis treatment.

## Introduction

Sepsis is a life-threatening organ dysfunction syndrome caused by a dysregulated host response to infection (Eisen et al., 2021). According to the recent Global Burden of Disease Study, an estimated 48.9 million septic cases occurred globally in 2017, with a mortality rate of 22.5%, constituting nearly 20% of all global deaths (Rudd et al., 2020). Without timely and effective interventions, the mortality rate can quickly surpass 30-35% (Abdul-Aziz et al., 2016). Apart from the high health-associated burden, septic shock incurs an estimated annual healthcare cost of $24 billion (Liu et al., 2022). The treatment of sepsis primarily encompasses three aspects: recognizing that infection is an underlying cause of sepsis, which also contributes to the onset and perpetuation of immune dysregulation; implementing infection control measures (such as antibiotics and source removal) for all septic patients, particularly in cases of circulatory shock, where hemodynamic management (fluid resuscitation and vasoactive agents) is essential; and modulating the host response (utilizing interventions such as vasopressin, hydrocortisone, blood purification, activated protein C/thrombomodulin, etc.), in conjunction with currently available intervention measures, primarily for patients experiencing septic shock (Stocker et al., 2017, Evans et al., 2021; Philips et al., 2021; Hagel et al., 2022). Additionally, some patients may require organ support, such as mechanical ventilation or kidney replacement treatment (Fowler et al., 2019; Hagel *et al.,* 2022). There is an urgent need to explore novel approaches for diagnosis and therapy. 

In the early stages of a systemic inflammatory response, immune balance can be rapidly restored when the immune system eliminates the pathogen in a timely manner (Siriwardena et al., 2022). However, if the pathogen is not eliminated promptly, it can lead to an imbalanced immune modulation, making patients susceptible to secondary infections (Svendsen et al., 2023). Both the innate and adaptive immune systems are capable of releasing inflammatory cytokines during the early stages of sepsis, effectively clearing foreign pathogens (Peters-Sengers et al., 2022). Innate immune cells play a crucial role in recognizing pathogens or pathogen-associated molecular patterns (PAMPs) (Lasola et al., 2021). With the release of C3a and C5a, the complement system can be activated simultaneously, representing a remarkable trait that leads to the release of damage-associated molecular patterns (DAMPs) (Moriyama and Nishida, 2021).

High mobility group box 1 (HMGB1) can be produced by dead or dying cells, facilitating the activation of innate immune cells and the release of cytokines (Yang et al., 2022).Subsequently, the excessive release of proinflammatory cytokines such as interleukin-6 (IL-6) contributes to organ failure, tissue injury, immunodeficiency, and other complications (Carestia et al., 2019).Overall, the excessive activation of inflammation and cytokine storms during the septic process remains the main causes of high mortality (Root-Bernstein,2021).Both innate immune dysfunction and acquired immune suppression may lead to multiple organ injuries and sepsis-associated deaths (Hensler et al., 2022).Therefore, it is crucial to maintain the balance between inflammation and anti-inflammation and ensure the proper functioning of both innate and acquired immune functions.

Long non-coding RNAs (LncRNAs) are a type of noncoding RNA, consisting of more than 200 nucleotides and lacking protein coding capacity (Sun et al., 2020; Wang Z et al., 2022). They have emerged as essential players in almost all levels of gene functions and regulation, being associated with a plethora of cellular functions (Ju et al., 2022). Recently, an increasing number of lncRNAs participating in the onset and progression of sepsis have been identified, such as metastasis-associated lung adenocarcinoma transcript1 (MALAT1) (Yue et al., 2022), cancer susceptibility candidate gene 2 (CASC2) (Wang R *et al.,* 2022), and noncoding RNA activated by DNA damage (NORAD) (Xie et al., 2022).Evidence has demonstrated that the lncRNA paternally expressed 13 (Peg13) plays a protective role against neurological diseases. Peg13 attenuates hypoxic-ischemic brain injury in neonatal murine models by binding to miR-20a-5p to elevate X chromosome-linked inhibitor of apoptosis (XIAP) expression (Gao et al., 2022). Peg13, induced by GLI Family Zinc Finger 2 (Gli2), reduces cerebral ischemia/reperfusion damage via the down-regulation of Yy1 in a PRC2 complex-dependent manner (Li et al., 2023). Peg13 ameliorates sevoflurane toxicity against neural stem cells by up-regulating SRY-box transcription factor 13 (Sox13) as a sponge for miR-128-3p (Jiang et al., 2020). Peg13 suppresses the course of epilepsy in murine models by inactivating Wnt/β-catenin signaling through the modulation of miR-490-3p/26S proteasome non-ATPase regulatory subunit 11 (Psmd11) (Feng et al., 2020).

In the current study, we hypothesize that Peg13 also participates in the inhibition of the systemic inflammatory response in sepsis. Our results show that Peg13 can alleviate the inflammatory response by reducing the release of proinflammatory cytokines HMGB1 and IL-6 in lipopolysaccharide (LPS)-induced cell and murine models. Clinically, Peg13 exhibited a remarkable up-regulation in the blood of septic patients and was negatively correlated with HMGB1, C-reactive protein (CRP), and erythrocyte sedimentation rate (ESR). Hence, Peg13 might be a potential therapeutic target for attenuating the inflammatory response in sepsis.

## Materials and Methods

### Cell culture

Human embryonic kidney cells 293T (obtained from the Shanghai Institute of Biological Sciences cell bank) were cultured in Dulbecco’s Modified Eagle Medium (DMEM; Thermo Fisher Scientific) supplemented with 10% fetal bovine serum (FBS) and 1% antibiotic (Thermo Fisher Scientific). The cells were maintained in a humidified incubator with 5% CO2 at 37 °C.

### Lentivirus infection and treatment

The complete culture medium was used to prepare cell suspensions at a concentration of 4 × 10^^4^ cells/mL, and 100 μL of the suspension was inoculated into a 96-well culture plate. The cells were then incubated at 37 ℃ for 16-24 hours until reaching 20-30% confluence. RNA interference sequences targeting LncRNA Peg13 were designed as follows: Control:5’-TTCTCCGAACGTGTCACGT-3’, Sequence1: 5’-GCGTGTTTACCCTGTGAATGG-3’, Sequence2: 5’-GCGTCATTACTTTGTGCATAG-3’, Sequence 3:5’-GGACATGAGCTGGCATCTTTC-3’. The short hairpin RNA (shRNA) and negative control (NC) of PEG13 were synthesized by Shanghai Genechem Co., Ltd. The lentivirus of LV-PEG13-RNAi (Shanghai Genechem Co., Ltd.) or LVCON313 negative control (hU6-MCS-CBh-gcGFP-IRES-puromycin) was successively diluted in complete culture medium to a final titer of MOI=10 (1 × 10^7^ TU / mL), MOI=50 (5 × 10^7^ TU / mL), and MOI=100 (1 × 10^8^ TU / mL). After removing the cell supernatant, the culture medium was exchanged under different conditions: i). 90 μL complete culture medium + 10 μL lentivirus with MOI=10, 50 or 100; ii). 86 μL complete culture medium + 10 μL lentivirus with MOI=10, 50 or 100 + 4 μL infection enhancement reagent HiTrans G A (Genechem); iii). 86 μL complete culture medium + 10 μL lentivirus with MOI=10, 50 or 100 + 4 μL infection enhancement reagent HiTrans G P (Genechem). Complete culture medium was replaced at 16 hours after infection. At 72 hours after infection, the fluorescence intensity was observed to be relatively high under a fluorescence microscope, and the optimal conditions for lentivirus infection were determined. 293T cells were stimulated with 50 ng/mL LPS and subsequently infected with either sh-NC or sh-PEG13 lentivirus. After 16 hours, the cell supernatant was collected for subsequent assays.

### Real-time quantitative PCR

The total RNA extraction was performed using TRIzol reagent (Invitrogen), followed by reverse transcription into complementary DNA with the PrimeScript RT reagent kit (Sangon). Subsequently, real-time PCR was conducted using SYBR Premix Ex Taq (Sangon). Peg13 was synthesized by Sangon Biotech (Shanghai) Co., Ltd., and its primer sequence was as follows: 5’-gACCACgAACCgAAgAggAC-3’ (forward), 5’-CgCggggAAAAAAAATATCATgC-3’ (reverse). The expression of Peg13 in the supernatant or blood specimens was detected using the Roche LightCycler^®^ 480 System. The reaction conditions included pre-denaturation at 95 °C for 30 s, denaturation at 95 °C for 40 cycles of 5 s, annealing for 40 cycles at 50 °C for 30 s, extension for 40 cycles at 60 °C for 30 s, followed by 95 °C for 5 s and 60 °C for 60 s.

### Enzyme-linked immunosorbent assay (ELISA)

After centrifugation at 3000 rpm for 10 minutes,the supernatant from cells and blood specimens was collected. HMGB1 and IL-6 concentrations were determined using ELISA kits according to the manufacturer’s specifications. Specifically, the Enzyme-linked Immunosorbent Assay Kit SEA399Hu 96 T, designed for the organism species Homo sapiens (Cloud-clone Corp., Wuhan), was employed for HMGB1 in clinical blood specimens. The Human HMGB1ELISA KIT SEKH-0409 96T (Solarbio life sciences, Beijing) was utilized for HMGB1 in mice plasma and cell supernatant. The Enzyme-linked Immunosorbent Assay Kit SEA079Mu 96T and SEA079Hu 96T (Cloud-clone Corp., Wuhan) were used for IL-6 in mice plasma and cell supernatant. 

### Animal experiment

We initially housed 22 male BALB/c mice weighing approximately 25-30 g, which were randomly numbered (no. 1-22) using an ear tagger, During the first week, all mice were observed to have normal hair, diet, and activity. These mice were grouped and divided into four groups (n=5-6): the control group (nos. 18/19/20/21/22), the sepsis model group (nos. 1/2/3/4/5/6), the Peg13 silent group (nos. 7/8/9/10/11/12), and the Peg13 silent control group (nos. 13/14/15/16/17). Mice were anesthetized with chlorambucil at 25-50 mg/kg. Mice No. 1-17 were intraperitoneally injected with 15 mg/kg LPS, while mice No. 18-22 received an equal amount of saline. All mice were fasted with free access to water 10 hours before the injection and were allowed to eat and drink freely 1 hour after the injection. In the Peg13 silencing group (no. 7/8/9/10/11/12), the LV-Peg13-RNAi expression vector was injected via the tail vein to establish the Peg13 silencing group, The Peg13 silencing control group (no. 13/14/15/16/17) was injected with the negative control virus CON313 to create the Peg13 silencing control group. Two injections within three days, conducted from 9:00 am to 10:00 am to complete all mouse injections.

After the mock-up, seven observation indicators were assessed: mice appearance, voluntary awareness, voluntary activity, response to stimuli, presence of eye discharge, respiratory rate, and respiratory quality. Each indicator was categorized into five levels (0-4 points). The total score for each mouse was calculated by summing up the scores of the seven indicators, with the highest possible score being 28, and this maximum score was assigned to deceased mice. Mice exhibiting signs of mental lethargy, agitation, increased respiration and heart rate, abdominal distension, heightened oral and nasal secretions, erect hair, and a rectal temperature change exceeding 1 ℃ after the procedure were considered successful.

After successfully constructing the Peg13 silent mouse model and injecting the negative control virus CON313, blood specimens were collected from all mice through the orbital plexus at 3 days, 7 days and 14 days (from 9:00 AM to 10:00 AM). Previous experiments indicated no significant changes in serum IL-6 and HMGB1 levels at 0 hours,12 hours, 24 hours, so 3 days, 7 days and 14 days, which were selected for analysis. All 22 mice were alive at the time of blood collection on day 3. On day 7, mice #3 and #8 had died, and no blood specimens could be obtained, while the remaining 20 mice were alive. On day 14, mouse 22 had died, and no blood specimen could be obtained, while the remaining 19 mice were alive. The blood specimens were allowed to clot naturally at room temperature for 30 minutes, then centrifuged at 3000 rpm for 5 minutes, the supernatant was labeled and stored at -80 °C. The experimental procedure was approved by the Animal Ethics Committee of the Shaoxing People’s Hospital.

### Patients and blood collection

In total, 44 septic patients and 36 healthy participants were included in the current study conducted at Shaoxing People’s Hospital from January 2021 to December 2022. The inclusion criteria were as follows: Patients were diagnosed following the Surviving Sepsis Campaign: International Guidelines for the Management of Severe Sepsis and Septic Shock, 2016. Exclusion criteria were: 1) admission time < 24 hours; 2) Accompanied by active malignant tumor disease; 3) History of acute respiratory tract, gastrointestinal tract, urinary tract and other organ infections within the past 2 weeks; 4) Those who had taken immunosuppressants within the past 3 months. All septic patients were further grouped into Sepsis patients (n = 34) and Septic Shock patients (n =10) based on the occurrence of SS according to the Third International Consensus Definitions for Sepsis and Septic Shock. Meanwhile, healthy volunteers were selected as controls following specific inclusion criteria: (i) the age distribution was the same as that of septic patients; (ii) Those with a history of acute respiratory tract, gastrointestinal tract, urinary tract and other tissues and organs infection in the past 2 weeks were excluded. There were 27 males and 17 females in the sepsis group, aged (68.45±12.50) years; 18 males and 18 females in the non-sepsis group, aged (44.64±12.51) years, with no statistical difference in the gender distribution between the two groups (P>0.05). In the sepsis group, 8mL of fasting peripheral venous blood was collected on the day following admission, and in the control group, on the day of the physical examination. Of this, 4mL was used for HMGB1 content testing and 4mL for lncRNA Peg13 relative expression testing. Additionally, the demographic and clinical characteristics of the study population, including age, gender, primary site of infection, duration of antibiotic treatment, duration of vasoactive drug administration, duration of corticosteroid use, Sequential Organ Failure Assessment (SOFA) score, C-reactive protein (CRP), procalcitonin (PCT), white blood cell count (WBC), erythrocyte sedimentation rate (ESR), interleukin 2(IL-2), interleukin 4(IL-4), interleukin 6(IL-6), interleukin 10(IL-10), tumor necrosis factor(TNF-α), and interferon (IFN-γ), are summarized in Table (Table S1 and S2). The procedure gained approval of the Ethics Committee of the Shaoxing People’s Hospital (Approval No. 2020-117).

### Statistical analysis

The results were presented as mean ± standard deviation. Data analysis was conducted using GraphPad Prism 9.0.1. One- or two-way analysis of variance (ANOVA) was employed for comparing three or more groups. Student’s *t*-test was used for comparing data between two groups. Pearson correlation test was performed to evaluate the relationships between Peg13 expression and HMGB1, CRP and ESR among septic patients. A significance level of P<0.05 was considered statistically significant.

## Results

### Peg13 presents the up-regulation in supernatant of LPS-induced 293T cells

**Figure 1 -:**
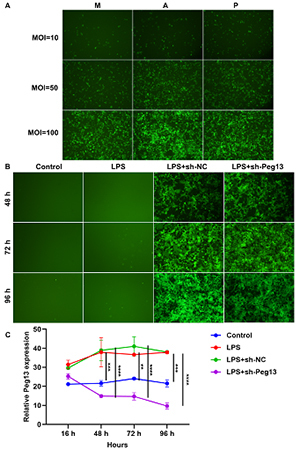
Peg13 Up-regulation in LPS-induced 293T cell supernatant silenced by shRNA Lentivirus (sh-Peg13). (A) Fluorescent images of 293T cells infected with 10 μL sh-Peg13 lentivirus (MOI=10, 50 or 100) along with (M) 90 μL complete culture medium; or (A) 86 μL complete culture medium and 4 μL HiTrans G A; or (P) 86 μL complete culture medium and 4 μL HiTrans G P at 72 h. (B) Fluorescent images of 293T cells exposed to 50 ng/mL LPS and infected with sh-NC or sh-Peg13 lentivirus at 48, 72, and 96 h. (C) Peg13 expression in 293T cells under control, LPS, LPS + sh-NC lentivirus or LPS + sh-Peg13 lentivirus conditions at 16, 48, 72, and 96 h. **p<0.01; ***p<0.001; ****p<0.0001.

This study established 293T cells induced with 50 ng/mL LPS. Lentivirus -mediated sh-Peg13 was then introduced into the 293T cells, and based on fluorescence intensity, the optimal infection condition was determined as 10 μL sh-Peg13 lentivirus with MOI=50,along with 86 μL complete culture medium and 4 μL HiTrans G P (Figure 1 A ). Additionally, after infection for more than 48 hours, the fluorescence intensity remained high (Figure 1 B ). In comparison to control 293T cells, Peg13 exhibited a significant up-regulation after exposure to LPS (Figure 1 C ). Following the infection with sh-Peg13 lentivirus for 48, 72, and 96 hours, Peg13 expression was notably attenuated in LPS-induced 293T cells.

### Peg13 suppression aggravates the release of LPS-induced proinflammatory cytokines HMGB1 and IL-6 in 293T cells

**Figure 2 -:**
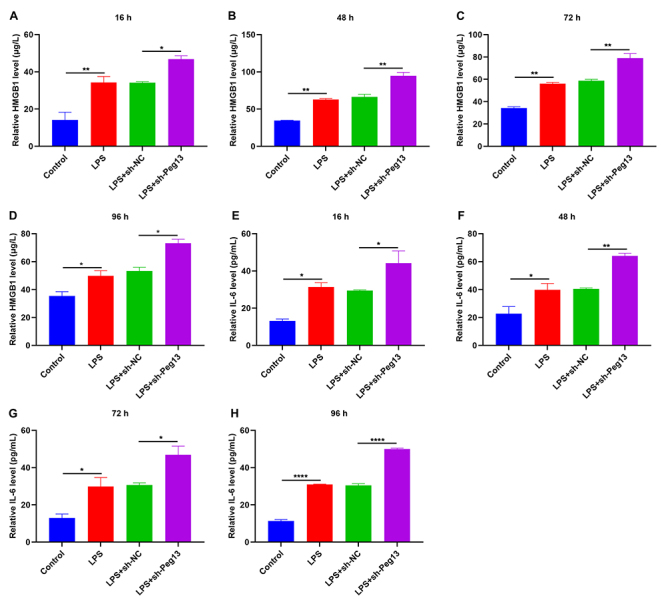
Peg13 suppression aggravates LPS-induced proinflammatory cytokines HMGB1 and IL-6 release in 293T Cells. (A-D) HMGB1 content in 293T cells treated with 50 ng/mL LPS and infected with sh-NC or sh-Peg13 lentivirus at 16, 48, 72, and 96 h. (E-H) IL-6 level in 293T cells exposed to 50 ng/mL LPS or/and infected with sh-NC or sh-Peg13 lentivirus at 16, 48, 72, and 96 h. *p<0.05; **p<0.01; ****p<0.0001.

In LPS-induced 293T cell supernatant, the proinflammatory cytokine HMGB1 was significantly up-regulated compared to that in the control supernatant (Figure 2A-D). During the infection with sh-Peg13 lentivirus for 16, 48, 72, and 96 hours, the HMGB1 level was further elevated in the supernatant of LPS-induced cells. Additionally, a higher level of IL-6 was observed in the LPS-induced cell supernatant compared to controls (Figure 2 E  -H). Following infection for 16, 48, 72, and 96 hours, the sh-Peg13 lentivirus further increased the IL-6 level in the supernatant of LPS-exposed cells.

### Peg13 expression is up-regulated in blood of septic mice

**Figure 3 -:**
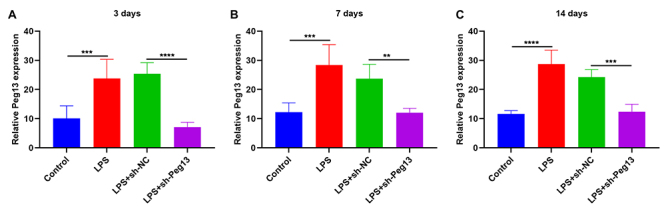
Peg13 expression is up-regulated in septic mice blood and its decrease by sh-Peg13 Lentivirus. (A-C) Peg13 expression in the blood of control mice, LPS-induced septic mice, and septic mice injected with sh-NC or sh-Peg13 lentivirus at 3, 7, and 14 days. **p<0.01; ***p<0.001; ****p<0.0001.

We divided BALB/c mice into the control group, LPS group, LPS + sh-NC group or LPS + sh-Peg13 group. Control mice were intraperitoneally injected with the same amount of saline, while mice in the remaining groups were intraperitoneally injected with 15 mg/kg LPS to induce sepsis. Mice in the LPS + sh-NC group or LPS + sh-Peg13 group were injected with sh-NC or sh-Peg13 lentivirus into the tail vein twice within three days. Peg13 exhibited higher expression in the blood of LPS-induced septic mice compared to that of control mice (Figure 3 A  -C). Blood samples were collected on days 3 (3 days 0 hours, 3 days 12 hours, 3 days 24 hours), 7, and 14 post-injection of sh-Peg13 lentivirus, resulting in a significant reduction in the expression of Peg13 in the bloodstream of septic mice.

### Peg13 inhibition exacerbates proinflammatory cytokines HMGB1 and IL-6 in blood of septic mice

**Figure 4 -:**
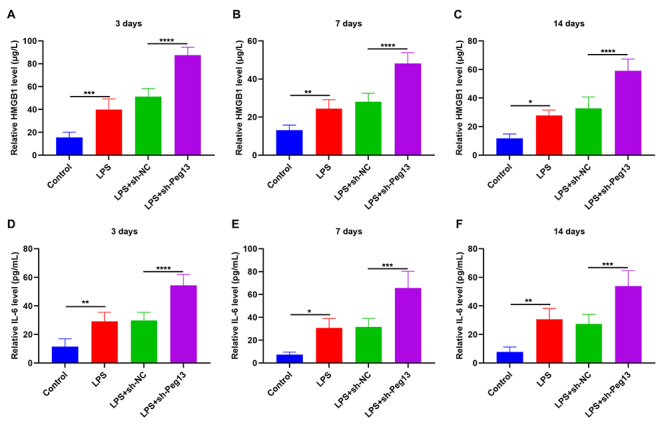
Peg13 Inhibition Exacerbates Proinflammatory Cytokines HMGB1 and IL-6 in Septic Mice Blood. (A-C) HMGB1 content in blood of control mice, LPS-induced septic mice, and septic mice injected with sh-NC or sh-Peg13 lentivirus at 3, 7, and 14 days. (D-F) IL-6 level in the blood of control mice, LPS-induced septic mice, and septic mice injected with sh-NC or sh-Peg13 lentivirus at 3, 7, and 14 days. *p<0.05; **p<0.01; ***p<0.001; ****p<0.0001.

Proinflammatory cytokines HMGB1 (Figure 4 A  -C) and IL-6 (Figure 4 D  -F) showed a significant up-regulation in the blood of LPS-induced septic mice compared to that of control mice, indicating the activation of an inflammatory response. The levels of HMGB1 and IL-6 in the blood of septic mice were further elevated on days 3, 7, and 14 following lentivirus transfection. Thus, the suppression of Peg13 further exacerbated the inflammatory response in the peripheral blood of septic mice.

### Peg13 and HMGB1 are up-regulated in septic patients’ blood and Peg13 negatively correlates to HMGB1, CRP and ESR

**Figure 5 -:**
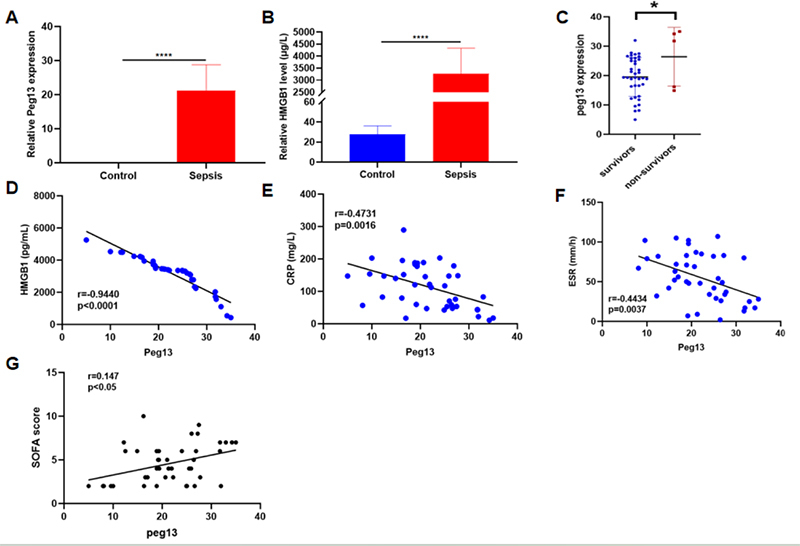
Up-regulation of Peg13 and HMGB1 in the blood of septic patients and negative correlation of Peg13 with HMGB1, CRP and ESR. (A) Peg13 expression in blood from healthy subjects and septic patients. (B) HMGB1 level in blood from healthy subjects and septic patients. (C) Peg13 expression is different between survivors and non-survivors. (D-F) Pearson correlation of Peg13 expression with HMGB1, CRP and ESR among septic patients. (G) Pearson correlation of lncRNA Peg13 expression with clinical SOFA score in patients with sepsis. *p<0.05; **p<0.01; ***p<0.001; ****p<0.0001;****p<0.0001.

A total of 44 septic patients and 36 healthy subjects were enrolled in our study, and their whole blood specimens were collected. Peg13 expression was detected in the blood of 43 septic patients (97.72%) (Figure 5 A ). Meanwhile, none of the healthy participants exhibited Peg13 expression in their blood specimens. In blood samples, HMGB1 levels in sepsis patients were higher compared to those in healthy participants (Figure 5B). We observed differences in Peg13 expression between the survivor and non-survivor groups (P<0.05) (Figure 5 C ). Among septic patients, Peg13 expression showed a negative correlation with HMGB1 (Figure 5 D ). It was also observed that Peg13 was negatively correlated with CRP and ESR (Figure 5 E , F). Besides the correlation with HMGB1, CRP and ESR, lncRNA levels were correlated with clinical SOFA score in these patients(P<0.05) (Figure 5 G ).

## Discussion

Sepsis, a systemic inflammatory response syndrome, remains a leading cause of death in intensive care units globally, particularly affecting the elderly population (Kamdar et al., 2022; Taylor et al., 2022; Zampieri et al., 2022). The mortality rate of sepsis has gradually decreased with the timely application of antibiotics (Richardson et al., 2022), fluid resuscitation (Zampieri *et al.,* 2022), and multiple organ support treatments (Kamdar *et al.,* 2022) over the past couple of decades. Despite these advancements, septic patients may succumb to the reactivation of the excessive inflammatory response triggered by the primary infection, either early or late in the course of the disease (Karimi et al., 2022 a ). Numerous studies have demonstrated that long non-coding RNAs (lncRNAs) are associated with diverse and complex cellular functions, playing crucial roles in sepsis, and they may serve as potential biomarkers and therapeutic targets (Han et al., 2021; Ma et al., 2021).

In this context, Peg13 was found to be up-regulated in lipopolysaccharide (LPS)-evoked cellular and murine models, as well as in the blood of septic patients, suggesting a potential role for Peg13 in sepsis.

HMGB1 is a ubiquitous nuclear protein found in nearly all cells, playing a role in modulating innate immune responses both within cells and in extracellular environments (Li et al., 2022). It plays a crucial function in pathological and pathophysiological processes, particularly inflammation (Werder et al., 2022). Aberrant systemic inflammatory responses are characteristic of sepsis, and reducing HMGB1 has been shown to alleviate inflammation and improve patient survival (Karimi et al., 2022 b ). Furthermore, HMGB1 in serum and urine has demonstrated excellent diagnostic efficacy for sepsis-triggered acute kidney damage (Zang et al., 2022). Our evidence confirms the up-regulation of HMGB1 in the supernatant of LPS-evoked cell models, blood of LPS-evoked murine models, and septic patients. 

Several compounds have shown potential as drugs against sepsis by targeting HMGB1. For example, Glycyrrhizin has the ability to alleviate sepsis-triggered acute respiratory distress syndrome by down-regulating HMGB1 and inhibiting the generation of neutrophil extracellular traps (Gu et al., 2022). Ulinastatin, in combination with Thrombomodulin, attenuates LPS-induced liver and kidney damage, partly through the modulation of HMGB1 (Zhang et al., 2022). Additionally, several lncRNAs participate in the onset and progression of sepsis by post-transcriptionally modulating HMGB1. Silencing nuclear enriched abundant transcript 1 (NEAT1) antagonizes LPS-triggered acute injury and inflammation in alveolar epithelial cells through the HMGB1/receptor for advanced glycation end products (RAGE) pathway (Zhou et al., 2020). Plasmacytoma variant translocation 1 (PVT1) induces M1 macrophage polarization and accelerates sepsis-induced myocardial damage through the miR-29a/HMGB1 pathway (Luo et al., 2021). Growth arrest specific 5 (GAS5) exacerbates myocardial depression in murine models with sepsis through the miR-449b/HMGB1 and NF-κB pathways (Gao et al., 2021), and reduces the inflammatory response of sepsis by attenuating HMGB1 release through the miR-155-5p/SIRT1 signaling (Zeng et al., 2023). HOX transcript antisense RNA (HOTAIR) aggravates kidney damage induced by urine-induced sepsis via miR-22/HMGB1 pathway (Shen et al., 2018). In the current study, Peg13 suppression improved the release of HMGB1 in LPS-induced 293T cells and murine models. Additionally, Peg13 exhibited a negative correlation with HMGB1 in septic patients. suggesting that Peg13 has the ability to modulate HMGB1 release during sepsis.

IL-6 plays a pivotal role in host defense against infection and tissue damage and serves as a bioindicator of cytokine storms (Kang and Kishimoto, 2021). The diagnostic and prognostic significance of IL-6 in sepsis has been extensively demonstrated (Song et al., 2019). Moreover, an elevated plasma IL-6 level is identified as a risk factor for intensive care unit-acquired weakness, affecting over 90% of patients with severe sepsis (Zanders et al., 2022). Several lncRNAs have been identified as regulators of IL-6 in sepsis, including NF-κB interacting lncRNA (NKILA) (Li et al., 2022), DILC (Huang et al., 2018), and MALAT1 (Liu et al., 2020). Silencing Peg13 exacerbated the release of IL-6 in both septic cellular and murine models. 

In addition to the acute inflammatory mediator HMGB1, Peg13 exhibited a negative correlation with CRP and ESR among septic patients. In the *in vitro* LPS-treated sepsis model, Peg13 expression correlated with the expression levels of HMGB1 and IL-6. These findings suggest that LncRNA Peg13 may also play a role in the non-specific inflammatory response by interacting with specific regulatory molecules, influencing the expression of CRP, HMGB1, and other acute-phase inflammatory response proteins. Further investigations are needed to explore the detailed mechanisms by which Peg13 participates in the development of sepsis.

## Conclusion

In summary, our findings strongly indicate that the long non-coding RNA Peg13 has the capability to mitigate inflammatory responses in both LPS-induced septic cells and murine models by reducing the release of proinflammatory cytokines HMGB1 and IL-6. Clinically, Peg13 expression was significantly elevated in blood specimens from septic patients and showed negative associations with HMGB1, CRP, and ESR among this patient group. Thus, Peg13 emerges as a potential therapeutic target for attenuating inflammatory responses in the treatment of sepsis.

## Supplementary material

The following online material is available for this article:

Table S1 **-**
General data between sepsis and healthy control group.

Table S2 **-**
Patient basic information and experimental data.
