# Supplementary material for: Revealing the role of Peg13: A promising therapeutic target for mitigating inflammation in sepsis
Source: Genet Mol Biol. 2024 May 31;47(2):e20230205. doi: 10.1590/1678-4685-GMB-2023-0205 (PMC11151158; doi:10.1590/1678-4685-GMB-2023-0205)
Supplement: Table S1 - [file 1415-4757-GMB-47-02-e20230205-s1.pdf]

Genetics and Molecular Biology
https://doi.org/10.1590/1678-4685-GMB-2023-0205

Supplementary Material to: "Revealing the role of Peg13: A promising therapeutic target for mitigating inflammation in sepsis"

Table S1 - General data between sepsis and healthy control group[n(%)].

**Features**	**Sepsis patients** （N=34）	**Septic Shock patients** （N=10）	**Healthy controls** (N=36)	***P* value**
Age (mean±SD, year)	65.20±13.61	69.41±12.21	44.64±12.51	<0.0001
Gender				
Male/ female	19/15	8/2	18/18	
Primary site of infection				
Urinary tract	8（23.53%）	1（10%）		
Skin and soft tissue	5（14.71%）	1（10%）		
Lung/thoracic cavity	7（20.59%）	4（40%）		
Abdomen	3（8.82%）	1（10%）		
Blood system	9（26.47%）	1（10%）		
Bone	0（0）	1（10%）		
Other	2（5.88%）	1（10%）		
Unfavorable prognosis	1（2.94%）	4（40%）		
Duration of Antibiotic (mean±SD,day)	12.32±5.66	12.00±4.35		0.8686
Duration of vasoactive drug (mean±SD,day)	2.33±0.58	8.33±4.39		0.0451
Duration of corticosteroids (mean±SD,day)	13.71±8.40	35.00±4.24		0.0124
SOFA score(mean±SD)	6.90±1.59	3.91±1.88		<0.0001
CRP(mean±SD,mg/L)	115.1±68.58	97.73±61.24		0.4760
PCT (mean±SD,ng /mL)	26.18±34.79	25.88±23.35		0.9796
WBC(mean±SD,×10^9 /L)	10.26±7.76	7.60±3.98		0.3049
ESR (mean±SD,mm/h)	58.85±36.41	52.40±29.37		0.6111
IL-2 (mean±SD,pg/mL)	6.59±18.76	4.35±4.49		0.7122

**Features**	**Sepsis patients** （N=34）	**Septic Shock patients** （N=10）	**Healthy controls** (N=36)	***P* value**
IL-4(mean±SD,pg/mL)	5.59±5.09	5.87±3.99		0.8733
IL-6 (mean±SD,pg/mL)	70.68±177.9	55.27±54.20		0.7897
IL-10(mean±SD,pg/mL)	12.61±13.69	24.81±40.49		0.1363
TNF-α(mean±SD,pg/mL)	10.31±28.48	5.18±3.408		0.5966
IFN-γ(mean±SD,pg/mL)	32.24±84.24	47.57±115.10		0.6464
