# Supplementary material for: Revealing the role of Peg13: A promising therapeutic target for mitigating inflammation in sepsis
Source: Genet Mol Biol. 2024 May 31;47(2):e20230205. doi: 10.1590/1678-4685-GMB-2023-0205 (PMC11151158; doi:10.1590/1678-4685-GMB-2023-0205)
Supplement: Table S2 - [file 1415-4757-GMB-47-02-e20230205-s2.pdf]

Genetics and Molecular Biology
https://doi.org/10.1590/1678-4685-GMB-2023-0205

Supplementary Material to: "Revealing the role of Peg13: A promising therapeutic target for mitigating inflammation in sepsis"

Table S2 - Patient basic information and experimental data.

No.	Sex	Age	CRP (mg/L)	PCT (ng/mL)	WBC (x10^9/L)	ESR (mm/h)	IL-2 (pg/mL)	IL-4 (pg/mL)	IL-6 (pg/mL)	IL-10 (pg/mL)	TNF-α (pg/mL)	IFN-γ (pg/mL)	HMGB1 (pg/mL)	Peg13
1	Female	60	148.57	29.18	5.84	37	2.03	10.67	122.5	30.82	4.99	6.87	2252.34	27.74
2	Male	34	47	0.14	6.69	9	1.65	2.17	8.52	2.37	2.17	4.7	3444.37	21.3
3	Female	56	112.5	9.42	8.66	85	2.42	2.51	32.06	8.19	2.84	6.15	3371.05	22.23
4	Male	69	203	14.04	12.87	34	1.8	1.56	65.44	6.55	1.83	9.48	3365.17	24.01
5	Female	49	47.68	0.38	6.36	26	111.64	<1.56	<1.81	1.35	<1.27	452.84	3091.13	26.6
6	Female	67	189.12	82.1	26.24	69	0.53	0.26	19.99	10.04	0.24	1.24	3471.10	20.66
7	Male	62	289.56	0.33	1.98	72	<1.52	<1.56	29.43	1.14	<1.27	1.59	4177.48	16.57
8	Male	79	44.14	6.83	13.51	80	<1.52	<1.56	58.41	9.98	<1.27	13.72	2032.27	31.77
9	Male	71	82.5	0.25	8.56	32	<1.52	<1.54	6.72	0.65	<1.27	<1.17	4496.34	12.19
10	Male	76	42.72	94.92	8.8	13	7.37	6.59	79.35	20.41	9.97	38.88	1735.05	31.79
11	Female	61	180.3	84.14	14.74	98	1.96	1.93	44.86	14.51	2.1	6.37	3629.04	19.32
12	Male	54	121.96	4.89	6.2	48	1.05	2.09	3.67	1.87	1.33	3.17	3407.56	21.92
13	Femal	61	59.81	0.43	8.9	7	1.36	2.09	43.25	20.5	2.17	18.47	3690.60	19.16

No.	Sex	Age	CRP (mg/L)	PCT (ng/mL)	WBC ($x10^9$/L)	ESR (mm/h)	IL-2 (pg/mL)	IL-4 (pg/mL)	IL-6 (pg/mL)	IL-10 (pg/mL)	TNF-α (pg/mL)	IFN-γ (pg/mL)	HMGB1 (pg/mL)	Peg13
	e													
14	Male	67	53.48	12.3	3.92	34	1.05	3.86	4.63	2.84	0.64	1.93	2342.31	27.48
15	Female	75	145.64	>100	36.68	87	4.04	<1.56	7.54	2.5	<1.27	6.3	3457.56	21.02
16	Male	93	53.14	10.85	7.78	29	1.09	2.09	4.33	2.11	2.38	6.37	3318.86	25.49
17	Female	63	17	85	6.53	56	12.63	8.37	67.91	18.56	7.48	11.44	3950.47	17
18	Female	72	178.68	35.27	9.1	49	2.5	5.78	83.33	13.14	4.12	5.5	2787.38	26.9
19	Male	67	147.42	79.11	7.73	82	2.57	4.26	106.4	28.5	1.96	8.89	4487.68	12.5
20	Male	62	120.34	0.089	8.19	50	4.78	9.2	6.08	3.28	5.79	10.27	3839.82	18.87
21	Male	73	202.9	35.01	12.37	79	8.3	16.43	1051.52	17.89	5.47	205.33	4539.53	10
22	Male	68	140.24	12.9	9.8	42	3.78	6.46	175.05	10.12	6.53	11.94	4244.66	14.90
23	Male	69	22.16	9.6	3.4	17	5.63	8.17	13.02	11.55	8.15	13.73	1568.77	31.97
24	Male	67	75.75	41.86	9.05	83	4.1	7.68	12.38	6.34	5.4	4.65	2786.36	27.24
25	Female	62	117.32	0.69	6.06	42	3.78	5.7	6.06	6.2	4.44	3.8	3332.04	25.03
26	Male	63	195.44	2.11	6.93	63	5.69	9.76	126.08	9.48	5.84	34.73	4207.92	16.3
27	Male	67	183.43	5.29	12.1	71	5.87	13.29	59.86	6.97	5.47	20.4	3728.84	18.89
28	Male	63	177.65	46.39	10.83	102	4.58	13.02	9.38	9.54	5.47	5.98	3611.40	19.32
29	Male	91	147.65	31.63	7.78	>140	1.9	4.65	33.92	12.33	4.38	4.59	5253.62	5
30	Male	88	17.94	13.95	5.85	28	3.52	6.29	12.2	7.57	6.06	6.48	431.19	35
31	Female	76	151.67	0.46	9.03	105	3.05	9.87	69.02	7.42	6.21	10.72	4191.54	16.57
32	Male	56	79.6	34.86	4.96	52	16.27	13.44	58.95	15.86	7.93	374.01	4228.65	16.18
33	Male	72	83.21	0.89	6.74	25	2.73	8.22	63.41	23.58	6.84	64.72	1113.28	32.96

No.	Sex	Age	CRP (mg/L)	PCT (ng/mL)	WBC ($x10^9$/L)	ESR (mm/h)	IL-2 (pg/mL)	IL-4 (pg/mL)	IL-6 (pg/mL)	IL-10 (pg/mL)	TNF-α (pg/mL)	IFN-γ (pg/mL)	HMGB1 (pg/mL)	Peg13
34	Male	59	188.48	9.08	12.62	83	4.37	2.85	22.17	10.74	9.21	8.53	3935.58	18.87
35	Female	76	187.02	74.89	13.47	48	7.17	12	167.31	62	5.91	6.15	3498.85	19.39
36	Female	34	11.12	26.16	4.06	17	6.25	11.26	11.6	12.13	9.6	11.6	546.11	34.22
37	Male	80	42.6	6.51	7.55	82	2.38	4.51	34.72	13.65	5.48	10.2	3360.02	24.92
38	Female	82	147.63	>100	25.49	107	6.36	18.74	87.49	35.61	29.03	72.46	3202.89	25.87
39	Male	83	58.43	0.39	1.26	2	1.12	1.52	19.73	138.42	1.23	34.89	3138.53	26.44
40	Female	79	70	0.15	3.89	54	1.5	1.21	35.48	1.82	1.76	7.74	3200.78	25.99
41	Male	72	153.7	4.92	3.77	102	2.92	3.65	58.27	4.74	158.64	6.37	——	9.57
42	Female	71	56.82	39.03	28.37	67	1.36	1.42	17.66	6.31	1.22	1.24	——	8.1
43	Male	88	20.94	1.6	3.97	44	2.47	2.25	13.54	45.73	4.89	4.72	——	7.94
44	Female	75	74.11	0.81	6.25	33	0.54	1.08	2.51	1.38	0.6	0.28	——	——
